# The Grouping of Subjects in Home Science

**Published:** 1912-01-27

**Authors:** Hilda D. Oakley

**Affiliations:** Warden of King's College for Women.


### INSTITUTIONAL HOUSEKEEPING.

(Workers' Questions and Criticism of every kind will be welcomed.)

### The Grouping of Subjects in Home Science.

By Miss HILDA D. OAKLEY, M.A., Warden of King's College for Women.

The grouping of subjects in the Home Science course is of supreme importance. The requirements of exact knowledge are great, and not less essential is it that education should be humane in method and spirit. We can only overcome ancient prejudices by proving that the rule of reason is not less powerful for good here than elsewhere. The various subjects of the course are to be regarded as instruments for bringing the full force of intelligence into closest relation with those simple activities of life, whose very importance has helped to keep them so long under the sway of custom. Let us look in this light at the leading subjects of the one-year post-graduate course, or, for those who take the three-year course, the third year. They are Chemistry of the Household; Hygiene and Sanitary Science; Economics; and the Practical Domestic Arts, including "Kitchen Laboratory work," connecting these with Chemistry.

### The Place of Economics.

The first question will probably relate to the place of Economics, a subject which bulks largely in the work at King's College for Women, but which may to many seem strange in a domestic science course. From one point of view, as it has always seemed to some of us, economics is the subject that unifies the course, because it ought to show the students the *raison d'être* of the scheme by revealing the relation of the work of household administration to other branches of work, and by giving the history of the social conditions which have produced the general features of modern home life, no less than the development of the industries on which it partly depends, the conditions of trade, and so forth.

As an example may be taken the curious evolution by which the large household has changed its character from a comparatively self-contained centre to one mainly depending on outside industries. The history of domestic service is another interesting theme. The study of economics liberalises the whole outlook of domestic science, and illustrates its relation to all other branches of social service. The household becomes for the student one amongst many institutions.

It is seen that the science which is the strength of good work in the public institution may be our strength also in the smallest home, whilst there is on the other hand no institution which will not be better served if we carry into it something of the spirit developed in genuine home-life.

Hygiene has been looked upon by some as the pivot subject; and it is of course easy so to conceive the study of the conditions of health as to see it comprising all others. Scientifically, biology and physiology may be thought to lead up to it, and it becomes a "cultural" study when we consider its social and national bearings. The question of the relative importance of subjects, however, need not be decided. We are only approaching a single reality by different avenues, and it is for the good of the whole if each teacher is so enthusiastic as to believe his own avenue the most essential.

### The Meaning of Domestic Science.

Domestic science, to which the beginning of the movement is generally traced, has long meant a training consisting for the most part of practical work in cooking, laundry, and housewifery, including as much theory as was found essential to the practice.

It is, I know, superfluous to argue in the pages of The Hospital that this training ought to be associated with a study of the elements concerned, in food, and in washing and cleansing materials, as scientific as our present knowledge of the chemistry of these subjects makes possible. We are obviously doing no more than continue, and carry on to a higher stage, work the importance of which has now for some time been recognised. We are increasing tenfold the power of vision into the most necessary tasks of daily life by developing the brain behind the eyes. By asking for such studies a place in the university curriculum we emphasise their intellectual rank, and hope that this emphasis will be far-reaching in the world outside the universities, even outside education.

### Some Objections Met.

It seems surprising, yet it was perhaps to be expected, that about this point in the scheme criticism has raged most fiercely. The objections from the side of "practical housekeeping" to the addition of this knowledge, as needless, the alarm at the introduction of theory, and it may be speculation, into the very stronghold of practical life, will no doubt die down when it becomes possible to compare side by side the results of narrow aims with those of far-sighted views of training. The criticisms of the scientific objectors, dreading lest the name of science should be lightly appropriated in a sphere where exact knowledge is most difficult to obtain, and where the complexity of the subject is obscured by its apparent familiarity, are of more weight.

They can be met, we believe, by the careful work of truly scientific minds in this field, and are not sufficient grounds for deferring that very system of education, in connection with which the resolve and inspiration to research are most likely to be called forth.

